# Transformer and Attention-Based Architectures for Segmentation of Coronary Arterial Walls in Intravascular Ultrasound: A Narrative Review

**DOI:** 10.3390/diagnostics15070848

**Published:** 2025-03-26

**Authors:** Vandana Kumari, Alok Katiyar, Mrinalini Bhagawati, Mahesh Maindarkar, Siddharth Gupta, Sudip Paul, Tisha Chhabra, Alberto Boi, Ekta Tiwari, Vijay Rathore, Inder M. Singh, Mustafa Al-Maini, Vinod Anand, Luca Saba, Jasjit S. Suri

**Affiliations:** 1 School of Computer Science and Engineering, Galgotias University, Greater Noida 201310, India; vandana.soni80@gmail.com (V.K.); alok_katiyar@hotmail.com (A.K.); 2 Department of Biomedical Engineering, North Eastern Hill University, Shillong 793022, India; bhagawatimrinalini07@gmail.com (M.B.); sudip.paul.bhu@gmail.com (S.P.); 3 School of Bioengineering Research and Sciences, MIT Art, Design and Technology University, Pune 412021, India; mahesh.nehu.333@gmail.com; 4 Department of Computer Science and Engineering, Bharati Vidyapeeth’s College of Engineering, New Delhi 110063, India; siddgupta462@gmail.com; 5 Stroke Monitoring and Diagnostic Division, AtheroPoint™, Roseville, CA 95661, USA; rajvivs888@gmail.com (V.R.); drindersingh1@gmail.com (I.M.S.); vinodanand2010@gmail.com (V.A.); 6 Department of Information Technology, Bharati Vidyapeeth’s College of Engineering, New Delhi 110063, India; tishachhabra2702@gmail.com; 7 Department of Cardiology, University of Cagliari, 09124 Cagliari, Italy; albeboi@tiscali.it (A.B.); lucasabamd@gmail.com (L.S.); 8 Department of Computer Science, Visvesvaraya National Institute of Technology (VNIT), Nagpur 440010, India; ekta.tiwari03@gmail.com; 9 Allergy, Clinical Immunology and Rheumatology Institute, Toronto, ON M5G 1N8, Canada; dralmainioffice@gmail.com; 10 Department of Electrical and Computer Engineering, Idaho State University, Pocatello, ID 83209, USA; 11 Department of Computer Engineering, Graphic Era Deemed to be University, Dehradun 248002, India; 12 Symbiosis Institute of Technology, Nagpur Campus, Symbiosis International (Deemed University), Pune 440008, India; 13 University Centre for Research & Development, Chandigarh University, Mohali 140413, India

**Keywords:** coronary artery disease, IVUS, wall segmentation, DL, UNet, transformer, AI bias

## Abstract

**Background:** The leading global cause of death is coronary artery disease (CAD), necessitating early and precise diagnosis. Intravascular ultrasound (IVUS) is a sophisticated imaging technique that provides detailed visualization of coronary arteries. However, the methods for segmenting walls in the IVUS scan into internal wall structures and quantifying plaque are still evolving. This study explores the use of transformers and attention-based models to improve diagnostic accuracy for wall segmentation in IVUS scans. Thus, the objective is to explore the application of transformer models for wall segmentation in IVUS scans to assess their inherent biases in artificial intelligence systems for improving diagnostic accuracy. **Methods:** By employing the Preferred Reporting Items for Systematic Reviews and Meta-Analyses (PRISMA) framework, we pinpointed and examined the top strategies for coronary wall segmentation using transformer-based techniques, assessing their traits, scientific soundness, and clinical relevancy. Coronary artery wall thickness is determined by using the boundaries (inner: lumen-intima and outer: media-adventitia) through cross-sectional IVUS scans. Additionally, it is the first to investigate biases in deep learning (DL) systems that are associated with IVUS scan wall segmentation. Finally, the study incorporates explainable AI (XAI) concepts into the DL structure for IVUS scan wall segmentation. **Findings:** Because of its capacity to automatically extract features at numerous scales in encoders, rebuild segmented pictures via decoders, and fuse variations through skip connections, the UNet and transformer-based model stands out as an efficient technique for segmenting coronary walls in IVUS scans. **Conclusions:** The investigation underscores a deficiency in incentives for embracing XAI and pruned AI (PAI) models, with no UNet systems attaining a bias-free configuration. Shifting from theoretical study to practical usage is crucial to bolstering clinical evaluation and deployment.

## 1. Introduction

Cardiovascular disease (CVD) is a major cause of morbidity globally [1]. Based on data analysis, research shows a forecast of a 96.6% mortality rate over a century in the area of CVD [2]. The two primary sources of mortality are coronary artery disease (CAD) and acute coronary syndrome (ACS) [3]. Due to the accumulation of atherosclerotic plaque inside the coronary walls, the narrowing of arteries causes CAD [3,4]. This build-up of atherosclerotic plaque makes the arterial walls thickened and stiff [5].

**Figure 1:**
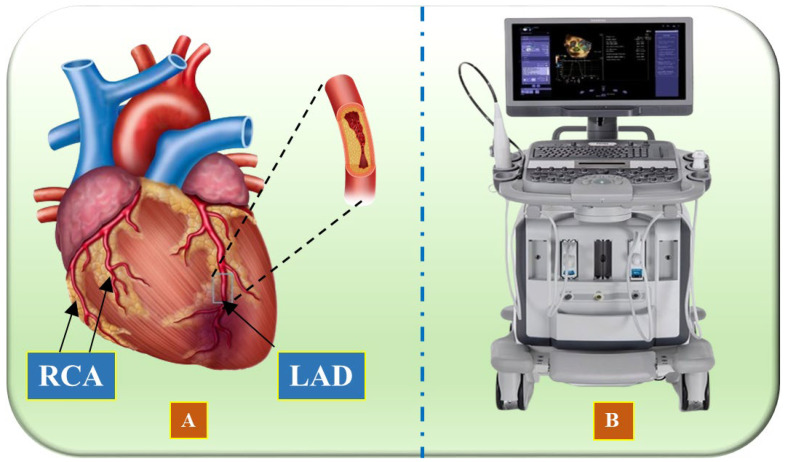
(**A**) Illustration of the coronary arteries of the heart, highlighting the LAD (left anterior descending coronary artery) and RCA (right coronary artery) (Image courtesy of Atheropoint™, Roseville, CA, USA). (**B**) IVUS acquisition device (Image courtesy of Dr. Alberto Boi and Luca Saba, University of Cagliari, Cagliari, Italy).

Vascular research and therapy have made significant progress over the last several decades, enhancing both the diagnosis and treatment of heart diseases [6,7,8,9]. Artificial intelligence (AI) solutions are enabled through the qualitative evaluation of morphology [10]. Several medical imaging modalities exist, namely, intravascular ultrasound (IVUS) [11,12,13], optical coherence tomography (OCT) [14,15], computed tomography (CT) [16], and magnetic resonance imaging (MRI) [17], which can be used to assist in identification, disease monitoring, and surgical planning [18,19,20]. An illustration of the coronary artery depicting the left anterior descending artery (LAD) and right coronary artery (RCA) is shown in Figure 1A, while the IVUS acquisition device is shown in Figure 1B.

IVUS stands out as a key modality [21], offering a unique perspective by allowing physicians to view coronary arteries internally [22]. Using a small ultrasound probe attached to a catheter, IVUS generates high-resolution, real-time images of the arterial walls [23]. This enables an accurate assessment of vessel size, plaque composition, and stenosis severity for treatment planning [24]. The process of lumen-intima (LI) and media-adventitia (MA) boundary detection is known as wall segmentation in IVUS scans or IVUS segmentation [25]. Accurate and automatic segmentation of coronary artery borders is vital for developing computerized systems to detect coronary artery stenosis and plaque. However, it faces several challenges. Some branches are too narrow for precise segmentation, and individual variations in the coronary artery tree add complexity. Additionally, similar looking vascular organs near the heart can be mistaken for coronary arteries. The coronary arteries occupy only a small portion of the heart, leading to segmentation imbalances. The limited availability of expertly labelled datasets further complicates the process. Since AI’s emergence, significant technological advancements have opened up numerous applications, including addressing these vascular segmentation challenges in medical imaging [26,27,28,29,30].

Machine learning (ML) approaches along with deep learning (DL) have greatly improved coronary artery segmentation by overcoming key challenges [31,32,33,34]. These techniques utilize large datasets (so-called big data) to detect complex patterns in IVUS images, enabling accurate and automated wall segmentation [35,36,37]. They reduce the need for manual intervention and subjective interpretation [38]. Additionally, ML and DL methods can adapt to various datasets and imaging conditions, making them versatile tools for wall segmentation in IVUS scans in different clinical settings [39]. In DL, solo DL (SDL) and hybrid DL (HDL) models both contribute to achieving high accuracy [40]. SDL models rely solely on neural networks trained on large datasets, while HDL models combine DL with other methods like rule-based systems or traditional ML to capitalize on their strengths [41,42]. For example, convolutional neural networks (CNNs) can be paired with conditional random fields (CRFs) [36,43] to enhance spatial segmentation. Hybrid models are effective in handling diverse medical imaging data, and by integrating handcrafted features with DL, they improve generalization and reduce overfitting, making them state-of-the-art [44].

Despite the significant advancements brought by DL models, including CNNs and hybrid approaches, previous state-of-the-art segmentation methods still face notable limitations. CNNs, while highly effective in feature extraction, are constrained by their limited receptive field, making them less capable of capturing long-range spatial dependencies in IVUS images. Additionally, deep CNN architectures require large, annotated datasets for robust training, yet IVUS datasets remain scarce and highly imbalanced, leading to biased segmentation outputs. Existing models also struggle with domain generalization, as variations in IVUS acquisition protocols and scanner types introduce challenges in adapting models across different datasets. Furthermore, computational complexity remains a barrier, particularly in clinical settings where real-time processing is crucial for intraoperative decision-making. Lastly, the black-box nature of many DL architectures raises concerns regarding explainability and clinical trust, making their direct adoption in healthcare settings challenging. These limitations underscore the need for more advanced architectures, such as transformer-based models, which offer superior global context understanding, improved generalization, and enhanced interpretability.

The integration of attention-based mechanisms into transformer architectures has led to significant improvements [45,46]. By harnessing the capabilities of attention and transformer-based models, wall segmentation algorithms in IVUS scans have attained greater precision and robustness [47], thereby enhancing diagnostic accuracy and facilitating more effective treatment strategies.

Consequently, we posit that transformer-based solutions outperform previously released models for segmenting walls in IVUS scans. While research on attention and the integration of transformers in DL architectures has grown steadily, no narrative review emphasizing transformers has been published. The proposed narrative review, utilizing the PRISMA model to select top publications, is a pioneering effort. This review offers (i) an overview of the primary categories of wall segmentation models in IVUS with illustrative examples, (ii) the architectures of these fundamental categories, and (iii) a thorough statistical analysis considering various AI aspects, such as segmentation processes, performance evaluation metrics, optimizers, loss functions, and learning rates.

The study is structured as follows: Section 2 details the PRISMA model, outlining the process of study selection and statistical analysis of the chosen articles. Section 3 provides a comprehensive background on the introduction to architectures for wall segmentation in IVUS and their classifications. Section 4 engages in a critical discussion, covering principal findings, recommendations, strengths, weaknesses, and potential extensions. Lastly, Section 5 concludes the review, summarizing the key insights and implications for future research.

## 2. Search Strategy and Statistical Distributions

We employed the Preferred Reporting Items for Systematic Reviews and Meta-Analyses (PRISMA) framework to systematically identify and analyze relevant studies in the domain of IVUS segmentation using DL and transformer-based models. The key terms utilized include DL, CVD, and transformer, along with specific phrases such as “IVUS segmentation”, “Coronary artery boundaries segmentation”, “IVUS segmentation using DL models”, “Lumen detection in IVUS scans”, “Media Adventitia detection in IVUS scans”, “Transformers in the coronary artery”, “Bias in DL/AI for CVD risk stratification”, “Attention in segmentation”, “IVUS segmentation using UNet”, and “Transformers in UNet”. To ensure comprehensive coverage, multiple academic databases—including Google Scholar, Science Direct, IEEE Xplore, PubMed, Springer, and Elsevier—were searched.

**Figure 2:**
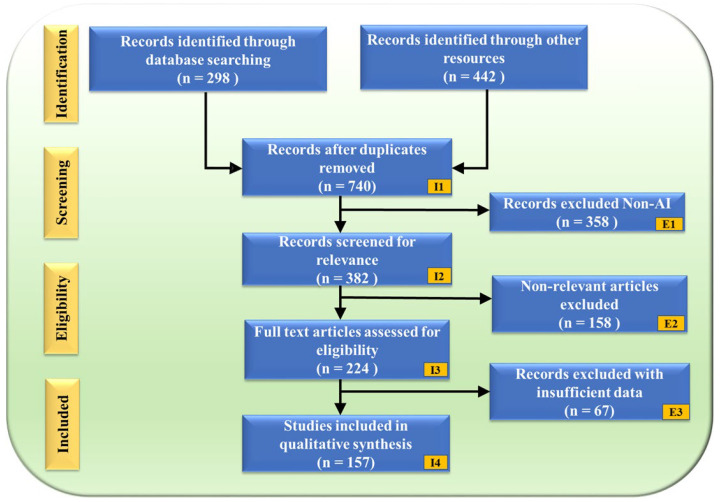
PRISMA model. “I” denotes Inclusion criteria, while “E” denotes Exclusion criteria.

The PRISMA flowchart (Figure 2) illustrates the study selection process, ensuring a structured and unbiased review. A total of 740 records were obtained after removing duplicates from an initial 298 database searches and 442 additional sources. These records underwent a rigorous screening process, where 358 non-AI-related studies were excluded (E1), followed by 158 non-genomic articles (E2). After full-text assessment, an additional 67 records were removed due to insufficient data (E3), resulting in a final selection of 157 studies for qualitative synthesis.

The selected studies were analyzed for their segmentation model architectures, performance metrics, optimization techniques, and biases within AI-driven IVUS segmentation. Additionally, the review explored the integration of transformer models into UNet architectures and examined their impact on segmentation accuracy and clinical relevance. A statistical analysis was conducted to assess the strengths and limitations of the identified approaches, shedding light on bias in AI systems and the role of explainable AI (XAI) in IVUS segmentation.

### 2.1. Statistical Distributions

**Figure 3:**
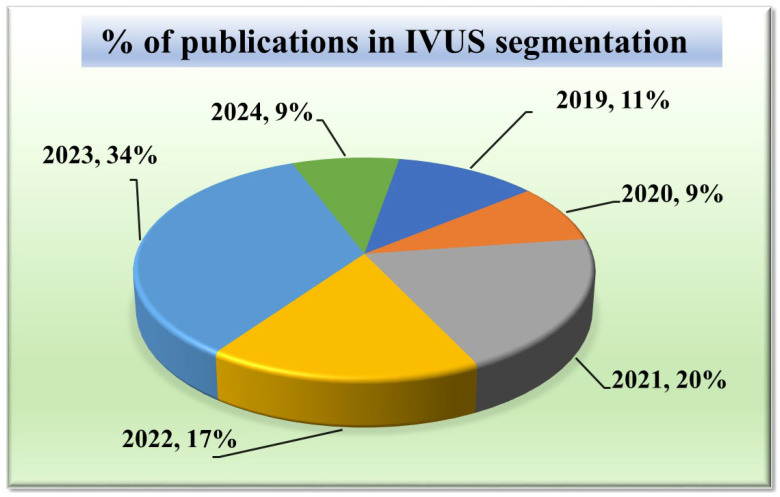
Publications in IVUS segmentation.

Given that our research focuses on segmentation techniques in IVUS scans, it is important to comprehend the current trends. Acquiring knowledge of the statistical distribution of the various segmentation techniques and their structures is essential to grasping the current state-of-the-art in this field. Furthermore, tracking the trend in publications over time might provide important insights into how IVUS segmentation techniques have changed and advanced. It can draw attention to how the performance and accuracy of these techniques are affected by technical developments, such as the addition of new algorithms or the integration of new architectures. Such statistical distributions of segmentation methods in IVUS frames are discussed in Figure 3.

#### 2.1.1. Publication Trend in IVUS Wall Segmentation

The chart in Figure 3 displays the number of research publications per year in the field of wall segmentation using IVUS from 2002 to 2024. It indicates growing research interest and progress in this area. The trend shows that 2021–2023 is the period where the highest amount of work was conducted in the field of IVUS. These potential spikes may correspond to significant advancements or new methodologies introduced such as attention and transformers in DL architectures.

#### 2.1.2. Publication Trend of Transformers in IVUS Segmentation

The integration of transformers within segmentation architectures [48,49,50,51,52,53,54] has emerged as a pivotal stride towards achieving unprecedented accuracy and yielding exclusive results. They use self-attention and hierarchical feature learning to understand the segmentation of IVUS images better, thereby improving the accuracy and reliability of segmentation.

#### 2.1.3. Distribution of Type of Transformers

**Figure 4:**
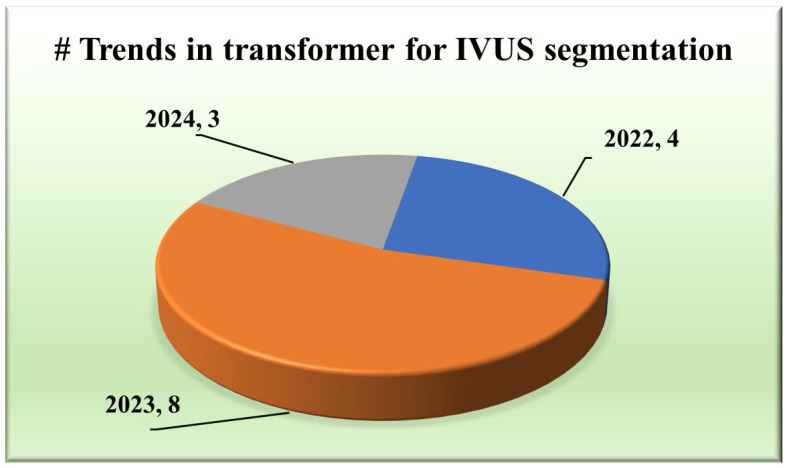
Trends in transformers for IVUS.

Various types of transformers, including the original transformer model, its variants, and more specialized architectures such as the UNet transformer, have been utilized in segmentation tasks. A gated axial attention layer has been introduced in medical transformers, which is used for building multi-head attention modules, increasing the receptive field of dilated transformers, etc. The introduction of vision transformers (ViTs) by Dosovitskiy et al. [55] in 2020 marked a significant milestone in adapting transformers for image segmentation tasks. However, in wall segmentation in IVUS, some specific architectures, namely the original (introduced in “Attention is All You Need”) [51,54], ViT [52], Cross-Stage Windowed Transformer (CSWin) [50], and Selective Transformer [49], have been found to be effective in capturing intricate features. Figure 4 illustrates this distribution.

#### 2.1.4. Distribution by Publishers

**Figure 5:**
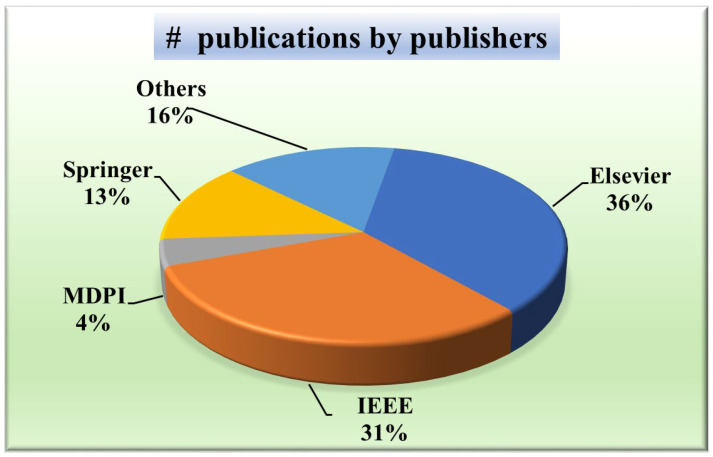
IVUS publications by publishers.

A wide distribution indicates a more holistic approach, considering perspectives from various publishers and potentially mitigating biases inherent in relying solely on a few sources. Knowing the publisher’s distribution will aid in evaluating the credibility and reliability of the research. For that purpose, Figure 5 presents a pie chart showing the distribution of publishers of papers taken into consideration. It depicts that the most common publisher is Elsevier (36%) [49,52,56,57,58,59,60,61,62,63,64,65], followed by IEEE (31%) [12,51,54,66,67,68,69,70,71,72,73,74,75,76], Springer (13%) [48,77,78,79,80,81], MDPI (5%) [31,82], SPIE (4%) [83,84], and the others constituted by PLOS [85], Wiley [86], Sage Publications [30], and ACC [87].

#### 2.1.5. Distribution of Evaluation Metrics

**Figure 6:**
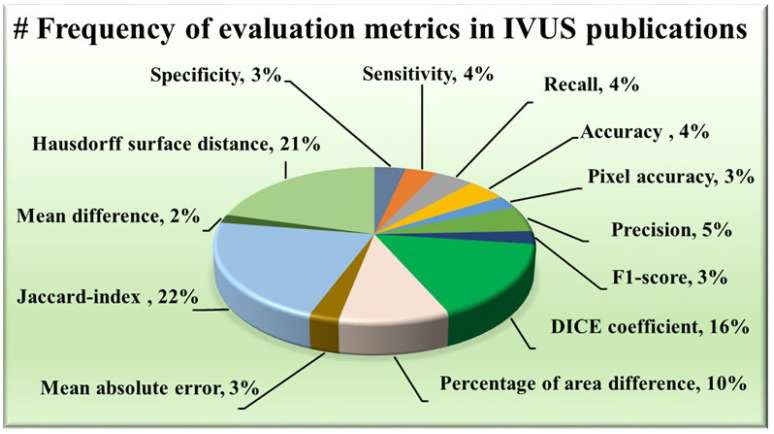
Frequency of evaluation metrics in IVUS publications.

The AI system has some statistical distributions and they play an important part in designing. These are attributes which serve as evaluation metrics for assessing the performance of various approaches, providing valuable insights into their effectiveness in delineating the structures of IVUS scans. Among the plethora of metrics available, certain trends have emerged regarding their prevalent usage in wall segmentation in the IVUS literature, as seen in Figure 6. Notably, the Hausdorff surface distance (HSD) [30,49,51,52,53,54,57,58,59,60,61,62,67,69,70,71,73,75,78,79,88], Dice coefficient [48,49,53,58,62,63,64,65,67,69,70,71,73,74,78,87,89], and Jaccard index [30,48,49,51,52,54,58,59,60,61,62,63,67,68,69,70,71,73,75,79,81,82,87,88,90] (also known as Intersection over Union) have consistently been favored due to their robustness and interpretability. The maximum distance from the segmented boundary point to the gold standard boundary nearest point is the HSD, providing insights into the surface dissimilarity. On the other hand, the Dice coefficient and Jaccard index measure the overlap between the segmented area and the ground truth area, with higher values indicating better agreement. Some other metrics have also been used namely, the percentage of area difference (PAD) [54,58,59,60,61,67,68,75,82,88], precision [48,53,68,74,76,80], and sensitivity [58,74,80,91] etc.

## 3. Classification of IVUS Segmentation Architectures

The walls of a coronary artery can be visualized in a cross-sectional view using IVUS imaging and are crucial to delineate for accurate disease diagnoses. Manual segmentation, while considered the gold standard, is time-consuming and laborious for cardiologists. Moreover, this method relies entirely on human subjectivity, making it prone to human errors and varying opinions. Therefore, there is a strong desire for the automated detection of intravascular borders, with researchers exploring various approaches in this ongoing research endeavor. Traditional image processing approaches [91], graph search [66], extremal regions selection [59], fuzzy system [92,93], and level set evolution [82,94] have come forward for this task. Wang et al. [88] introduced a feature selection method based on the Fractional-order Darwinian particle swarm optimization (FODPSO) algorithm. It is an intelligent optimization algorithm which provides better global search ability than PSO. These are categorized as conventional methods of segmentation since they typically rely on mathematical techniques rather than explicitly incorporating knowledge-based systems. Various ML algorithms have taken IVUS segmentation forward, offering diverse approaches to tackle its challenges. Supervised training methods [86,87] leverage labelled data to teach algorithms, while unsupervised techniques like K-means clustering [67] provide valuable insights by grouping similar image regions. Ensemble methods combine multiple algorithms [68], leveraging the strengths of each component and producing a powerful component. While traditional and ML techniques laid the groundwork, DL approaches [72] have revolutionized the field by overcoming several limitations. CNNs have demonstrated superior training efficacy compared to traditional ML algorithms due to their ability to process data in their raw form. The employ convolutional layers to extract patterns, eliminating the need for manual feature engineering and pooling layers for dimensionality reduction and abstraction. Furthermore, CNNs offer versatility in segmentation tasks through techniques like transfer learning. Pre-trained models such as InceptionV3 [95], DeepLabV3 [63], and ResNet [62] can be utilized for segmentation tasks. ResNet introduces a residual connection that mitigates the vanishing gradient problem, enabling smooth training of deeper networks. The primary objective of this review is to comprehensively analyze the four main categories of segmentation methods for IVUS boundaries that are instrumental in CVD risk stratification. Section 3 is organized into distinct sections, including (i) Conventional Techniques, (ii) ML Techniques, (iii) DL Techniques, and (iv) Attention and Transformer Methods. The DL techniques section is further subdivided into (i) Non-UNet methods and (ii) UNet and its Variants, providing a detailed exploration of each approach’s unique contributions and applications in the context of IVUS boundary segmentation for CVD risk assessment.

### 3.1. Conventional Methods

Earlier image processing has been a longstanding technique for segmenting structures within IVUS images. This involves applying various algorithms such as thresholding [60,96], active contours [56,58,96], fast marching method (FMM) [57] etc., to analyze the image data, identify edges, and differentiate tissue types based on intensity. Out of these, we have highlighted representative approaches such as thresholding and active contour models for segmenting walls in IVUS scans.

#### 3.1.1. Thresholding

**Figure 7:**
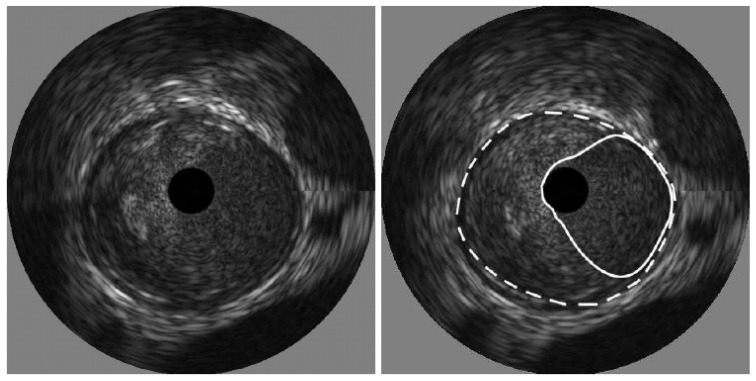
Detected boundaries of IVUS scan [70].

The process of thresholding involves selecting a threshold value and then classifying pixels in the image based on intensity values. In IVUS segmentation, the threshold value is chosen to differentiate between the lumen and the surrounding tissues, such as the vessel wall or plaque deposits. Kermani et al. [60] presented a ground-breaking nonparametric statistical method for precisely identifying media-adventitia (MA) and lumen-intima (LI) borders in ultrasound frames. Unlike traditional techniques, this approach offers an independent border extraction process at each cutting angle. By enhancing the conventional analysis of variance (ANOVA) criterion with a regularization term customized for IVUS images, the proposed method is designed to be versatile, fully automated, and suitable for parallel implementation. This process involves three steps, beginning with pre-processing the image. Here, a filter tackles the grainy speckle noise common in ultrasound data, while edge enhancement techniques like filtering or gradient calculations highlight the crucial borders. The second step involves dividing the image into small, overlapping windows, where each window pixel’s intensities are analyzed, potentially involving calculations like mean, standard deviation, or even more complex statistics. By scrutinizing the statistical information, the algorithm identifies the locations for both the lumen and MA borders within each window. Since initial detections can be noisy, morphological operations like erosion and dilation come into play in the final stage for refinement. The advantages of this method include its ability to effectively remove catheter artefacts, estimate borders sequentially, and provide an ad hoc mechanism for detecting and correcting discontinuous borders, ultimately providing a Jaccard measure of 0.84 ± 0.07 for Lumen, and 0.82 ± 0.11 for MA border. The results of the detected boundaries for seven images are shown in Figure 7 (white bold is lumen border and dotted border is the MA border).

#### 3.1.2. Active Contours

The active contour technique, often referred to as snakes, is a method used for segmentation tasks. It involves the delineation of boundaries within an image by iteratively deforming a parametric curve to fit the contours. The active contour model aims to minimize an energy functional E_snake_, which is the sum of internal energy E_int_ and external energy E_ext_.

E_snake =_ E_int_ + E_ext_

Internal energy represents the energy associated with the configuration of the snake itself. It encourages smoothness and regularity in the shape of the contour. External energy represents external forces acting on the snake. It guides the snake toward features of interest, such as boundaries or image gradients. Initialization is crucial as it provides the starting point for the snake’s evolution. The final contour represents the boundaries of the segmented areas.

Giannoglou et al. [56] introduced a fully automated method for segmenting the LI and MA using an active contour model. The model’s initialization leverages the inherent morphological characteristics of IVUS images through a two-step process: first, utilizing intensity information and then low-frequency information. This approach capitalizes on the adventitia’s representation as a thick echo-dense ring in IVUS images, which appears as a thick bright curve in polar coordinates. The study demonstrated a 96% reduction in analysis time compared to manual segmentation. Wang et al. [96] developed a framework for detecting the adventitia boundary by enhancing the traditional snake algorithm and incorporating Otsu thresholding, morphological operations, and connected component labeling.

**Figure 8:**
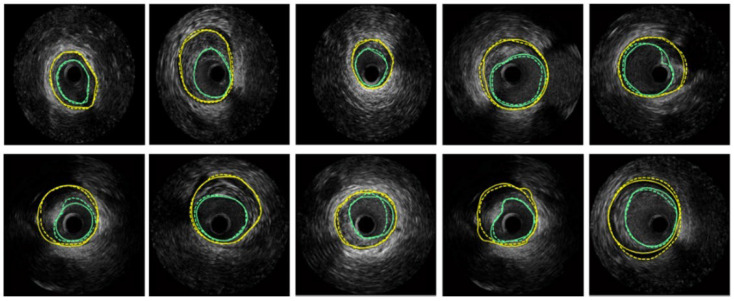
Segmentation of several IVUS frames [70], showing the lumen boundary (green) and media-adventitia (MA) boundary (yellow). Solid lines represent ground truth annotations, while dotted lines indicate predicted segmentations from the model.

Hammouche et al. [58] achieved significant success with a helical active contour algorithm based on the Rayleigh distribution of grey levels. This innovative algorithm is fast, employs an adaptive, simple, space curve for 3D lumen extraction, and is fully automated. It outperformed various traditional methods, achieving 98.9% accuracy. Figure 8 displays the segmentation results obtained on several IVUS frames, where turquoise color are the LI borders and yellow are the MA borders.

### 3.2. Machine Learning Techniques

The turning point came when traditional image processing methods struggled to handle the complexity and variability of IVUS images. Machine learning offered a more adaptable approach, allowing algorithms to learn from data and improve segmentation accuracy. Several methods emerged, including probabilistic approaches, contrast features analysis [84], acoustic shadowing detection [97], support vector machines (SVMs) [98], XGBoost (XGB) [99], and more, each contributing uniquely to improving segmentation accuracy and reliability. The following text emphasizes the use cases in Markov random fields (MRFs) [77] and K-means clustering [100,101].

#### 3.2.1. Markov Random Field

Probabilistic approaches employ statistical models to model the relationship between image features and tissue classes. These models assign a probability to each pixel belonging to a specific class [70]. This allows for a more robust segmentation compared to simpler thresholding techniques. Within the realm of probabilistic approaches for IVUS segmentation, MRFs offer a powerful technique for incorporating spatial context. It uses two types of potential functions: unary potentials that consider the observed data (like pixel intensities) to measure how likely a pixel belongs to a particular label, and pairwise potentials that capture spatial dependencies between neighboring pixels.

Optimization methods like graph cuts, belief propagation, or simulated annealing are employed to find the optimal labelling configuration that minimizes the overall potential energy. Further, MRFs were combined with the Rayleigh Mixture Model (RMM) by Gao et al. [85]. The RMM is utilized for pixel classification based on their gray levels. By leveraging the spatial relationships among neighboring pixels, it effectively clusters pixels. MRFs are employed for the angular place of calcified plaques detection, utilizing prior information derived from RMM results through a curve known as the maximum intensity curve (MIC). Through the MRF, the relationship of observed and hidden variables is computed, enabling the identification of calcified plaques with acoustic shadowing. Araki et al. [77] quantify the volume of calcium deposits within coronary artery walls by employing three different methods for soft computing: fuzzy c-means (FCM), K-means clustering, and hidden Markov random fields (HMRFs). These techniques essentially group pixels in the image based on their characteristics. Nevertheless, designing MRFs and defining appropriate potential functions can be challenging, and the computational cost may be high. As the technology advanced, ML models such as the random forest classifier and SVM captured the industry.

#### 3.2.2. Random Forest

Random forest is a ML algorithm that excels by combining the predictions of decision tree numbers. The trees are trained on random training subsets and features separately, which helps mitigate overfitting and enhances model robustness. This inherent randomness allows random forest to effectively capture non-linear relationships between features and segments. Moreover, random forest provides insights into feature importance, indicating which features contribute most significantly to the segmentation process. This feature analysis aids in understanding the underlying characteristics driving segment formation. It handles high-dimensional data well, automatically selecting relevant features for each tree, making it suitable for complex datasets and well-tried in CVD risk stratification using carotid plaque [102,103]. In 2017, Lo Vercio et al. [83] used random forests (RFs) to identify specific morphological structures within vessel walls seen in IVUS images. Later, in 2019, they built upon the concept of structure detection but took a more comprehensive approach by combining SVM with the RF model. First, they trained SVM to classify each pixel in the IVUS image [61]. Recognizing that the presence of structures like bifurcations or calcifications can challenge SVMs, the researchers then introduced RFs. These RFs are trained to identify such morphological structures within the image and refine boundaries. It was chosen based on its ability to deal with multiclass and imbalanced problems in association with randomness under sampling.

### 3.3. Deep Learning Techniques

Traditional methods often struggle with the complexity and variability of IVUS images, which are prone to noise, artefacts, and inconsistencies in tissue appearance due to factors like blood, calcifications, and imaging artefacts. However, the use of DL has overcome these challenges by effectively handling intricate patterns within the data [104]. DL algorithms can automatically learn to recognize and differentiate between subtle variations in tissue characteristics, reducing the impact of noise and artefacts, and improving the accuracy and consistency of image interpretation [105,106].

#### 3.3.1. Non-UNet Paradigms

The significance of CNNs in image segmentation lies in their ability to learn hierarchical representations of image features. They can automatically learn relevant features from the raw IVUS image during the training process by capturing low-level (edges, textures) and higher-level features that represent more complex patterns.

##### Scale Mutualized Perception

Existing DL methods struggle with scale-dependent interference due to their top-down aggregation of multi-scale features. To address this, Liu et al. [69] proposed scale mutualized perception, which considers scales that are adjacent together for the conservation of the complimentary outputs. Semantic cues are offered by the small scales for tissue localization and help in perceiving context globally to enhance context in large scales for local representation, and vice versa. By these, similar objects with local features can be distinguished. Detailed information was brought by large scales for vessel boundary refining. The architecture consists of three main components: (i) neighboring-scale interactive learning (NIL), (ii) scale-mutually context complement (SCC), and (iii) densely-connected atrous convolution (DAC). NIL refines blurriness by incorporating complementary features from adjacent scales, avoiding noise interference. SCC distinguishes objects with similar context appearances by transferring context between adjacent scales. It extracts global context from smaller adjacent scales and local context from larger adjacent scales using a non-local method. DAC employs a densely connected structure to reduce the loss of detailed information on vessel borders varying in size, large-scale to small-scale features merging, and enlarging the receptive field.

##### Combining Shallow and Deep Networks

Yuan et al. [73] introduced a two-facet structure called combining shallow and deep networks (CSDN) for better and more accurate segmentation. This framework targets low-level detail extraction with the help of thick channels, and the shallow network is responsible for learning high-level semantics. The framework is designed with five stages for extracting context and semantic information features, providing a large receptive field. The first step is the Stem block, which has two parts for different types of input downsampling. Following batch normalization and the PReLU activation function, the outputs are concatenated and convolution is performed. The Context Block, the last step, provides the maximal receptive field by embedding global contextual information using global average pooling and residual connections. Gather-Expansion Blocks make up the final three phases. The model provides great accuracy and efficiency for real-time segmentation by processing these data independently. A mutually guided fusion module is used to improve and fuse both kinds of feature representations in order to further improve segmentation performance.

#### 3.3.2. UNet Paradigms and Its Variants

UNet is a convolutional neural network architecture introduced in 2015 by Olaf Ronneberger [107] for biomedical image segmentation tasks. It has become the de facto standard for many image segmentation [108] problems due to its ability to capture precise spatial information and efficiency. It comprises an encoder (contract path) that captures context and spatial information through repeated convolutions and max pooling, a decoder (expansion path) that enables precise localization by combining upsampled output with high-resolution features and skip connections providing a seamless fusion of high-resolution features from the encoder with corresponding decoder layers, reserving spatial information. UNet is preferred nowadays as a base architecture because of its ability to effectively retain and combine low-level and high-level features through its skip connections, allowing for precise pixel-wise segmentation even with limited training data [109,110]. Numerous variants and specialized versions have emerged which fuse transfer learning-based UNet [111,112] to further optimize its performance and cater to specific issues [90].

##### MFA-UNet

**Figure 9:**
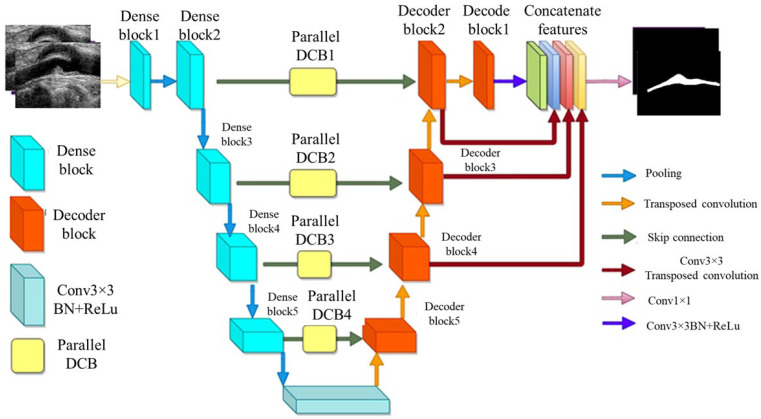
MFA-UNet’s architecture [115].

A variant of UNet, known as *multi-scale feature aggregated UNet* (MFA-UNet), integrates a feature aggregation module (FAM) incorporating convolutional bidirectional long short-term memory (BConvLSTM) units. With multi-scale inputs and careful supervision, this feature improves both the encoding and decoding stages by enabling context retrieval from spatial–temporal perspectives. The MFA-UNet’s whole architecture is seen in Figure 9. The FAM component was employed from BCDUNet [113], shown in Figure 9, to improve feature fusion and information retention. The factors, repeated from a relevant layer of encoders (skip connection), include local information of high resolution. In contrast, the factors received by the previous up-convolution layer provide the global semantic information. Information that was lost during cascaded encoding processes is restored by concatenation. ConvLSTM-type recurrent neural networks (RNNs) are capable of handling complicated item distributions, capturing spatiotemporal correlations of sequential input, and remembering previously learned information. Two ConvLSTMs are used to process both forward and backward input. The benefit of this network is that it includes multiscale inputs, the FAM module, and deep supervision of the UNet model to obtain adequate learning with a limited set of detailed IVUS scans. The Focal Tversky loss is used to optimize the MFA-UNet which addresses the issue of data imbalance [114].

##### IVUS-UNet++

The UNet++ model’s advanced system is called the IVUS-UNet++ model [116]. In UNet++, the encoder and decoder sub-networks are interconnected through a series of nested, dense convolutional blocks. The redesigned skip pathways aim to minimize the semantic gap between the feature maps of the encoder and decoder sub-networks. UNet++ effectively captures fine-grain details of foreground objects by gradually enriching high-resolution feature maps from the encoder network before fusing them with the corresponding semantically rich feature maps from the decoder network. Experimental results show that UNet++ with deep supervision achieves an average IoU (Intersection over Union score) gain of 3.9 points over the classical UNet [117].

**Figure 10:**
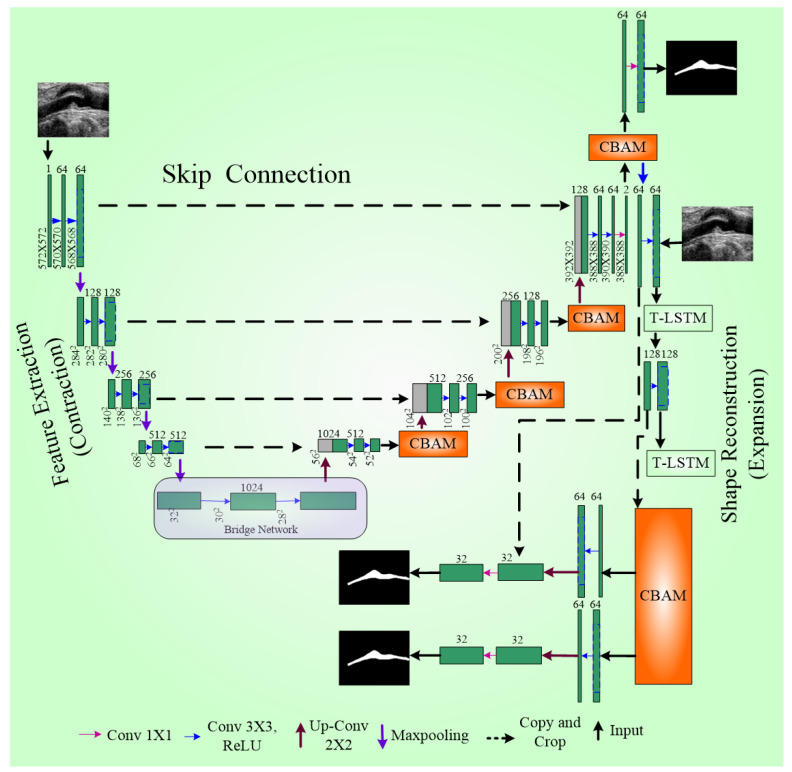
UNet++ architecture [118].

To further enhance the model, a pyramid of factors were integrated into UNet++, resulting in IVUS-UNet++. This addition enables the feature maps utility at different scales, effectively fusing and propagating feature information across various spatial scales throughout the network. Five types of feature maps supervise the feature maps present over the convolutional block (0.5), as illustrated in Figure 10. Upscale operators ensure size compatibility during the fusion of multi-scale features. A voting mechanism with parallel connection is used to generate the final probability map.

Several optimization strategies were employed to improve boundary detection. First, pre-trained weights were used as the backbone to mitigate the gradient vanishing problem in this deep network. Second, batch normalization (BN) was applied, and third, an activation function called ReLU was used consecutively with the convolution layer for network training improvement. Compared to other models, namely UNet++ [116] and IVUS-Net [79], IVUS-UNet++ achieved the best JM (Jaccard index) and HD (Hausdorff distance) for both the lumen and MA border. A schematic diagram of UNet++ is shown in Figure 10.

### 3.4. Attention and Transformer Methods

The NLP tasks were initially performed through a self-attention mechanism as suggested by Vaswani et al. [119], and the transformer (one form) played a major role in the field, being able to capture the decencies of long-term. Researchers started integrating attention [27,48,120,121,122] and transformers [17,46,123,124,125,126,127,128,129,130,131] into the medical field to enhance performance. Previously, image segmentation encountered numerous hurdles such as the inability to focus on relevant regions, limited contextual understanding, and challenges in dealing with objects of variable sizes. These shortcomings prompted a quest for innovative solutions, with attention mechanisms emerging as a promising avenue. The transformer model [119] is comprised of multiple encoder–decoder components, with each encoder having two parts: a feed-forward neural network and a self-attention layer. Each sublayer is followed by layer normalization and has a residual connection around it. Patch extraction breaks down the image into separate non-overlapping patches, flattened into a 1D vector. Each patch is then transformed into an embedding vector, potentially capturing features extracted by CNNs. Finally, in positional encoding, spatial information, both absolute and relative, is preserved to maintain the image’s spatial context. These vectors first flow through a self-attention layer, a layer that helps the encoder look at other words in the input sentence as it encodes a specific word. There, it computes the attention-infused representation:

$$Attention\left(Q,K,V\right)=softmax\left(\frac{QKT}{\sqrt{d_{k}}}\right)V$$

A feed-forward neural network receives the self-attention layer’s outputs. An extra attention layer in the decoder aids in concentrating on pertinent input segments. Finally, a linear layer projects the decoder output into a larger logit vector, whose probabilities are determined by a softmax layer. The word with the highest probability becomes the output for that time step. Recently, there has been a fusion of transformers with deep-learning architectures [50], notably combining CNNs and transformers, as exemplified by Tao et al. [51] This integration capitalizes on the complementary strengths of each; transformers capture remote dependencies while CNNs excel at local information extraction. It introduced transformer encoding blocks to model features extracted by CNNs at various scales, thereby enhancing both remote dependencies and local contexts [132]. Furthermore, they proposed a channel enhancement module employing a gated mechanism to enhance feature channels with more pertinent information.

#### 3.4.1. Perceptual Organisation-Aware Selective Transformer Framework

Sangala et al. [41] introduced a novel *perceptual organisation-aware selective transformer* (POST) framework designed to accurately segment vessel walls in IVUS images by mimicking the methods of cardiologists. Motion features with temporal context-based encoders are extracted, while a transformer module captures precise boundary information, guided by a boundary loss. The segmentation results are then made to combine a fusion module and temporal constraining (TF module) to obtain the correct boundaries. This framework has been integrated into software called QCU-CMS1 version 4.69 software (Leiden University Medical Center, Leiden, The Netherlands) for automatic IVUS image segmentation. The model predicts the boundaries that are missing by simulating the perceptual organization principles of human vision.

**Figure 11:**
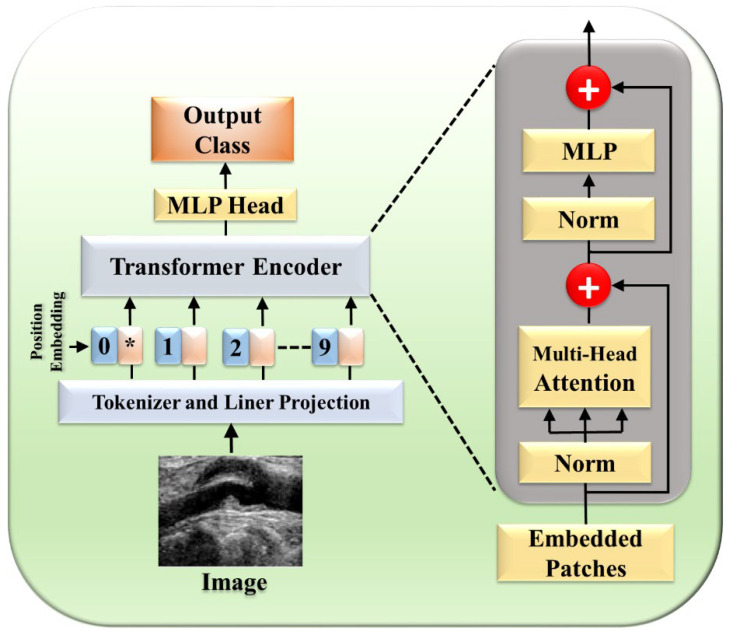
Selective STR UNet. The star (*) denotes the CLS token, which aggregates global feature information.

The framework consists of three major components (Figure 11): temporal context-based feature encoders, Selective Transformer Recurrent UNet (STR UNet) with a discriminator, and a TF Module. The temporal context-based feature encoders include encoders such as rotational alignment and visual persistence. The rotational motion of the vessel from the heartbeats was removed by the rotational alignment, while the residual visual factors in sequential order were captured by the visual persistence encoder. STR UNet can infer borders in dark regions by the use of generative adversarial learning (GAN) putting a specific loss function as a penalty during training. A discriminator is added to enhance the inference capability of STR UNet. The TF Module is a post-processing module that provides an improvement to the accuracy of annotation of side-branch and calcium areas.

#### 3.4.2. Multilevel Structure-Preserved Generative Adversarial Network (MSP-GAN)

**Figure 12:**
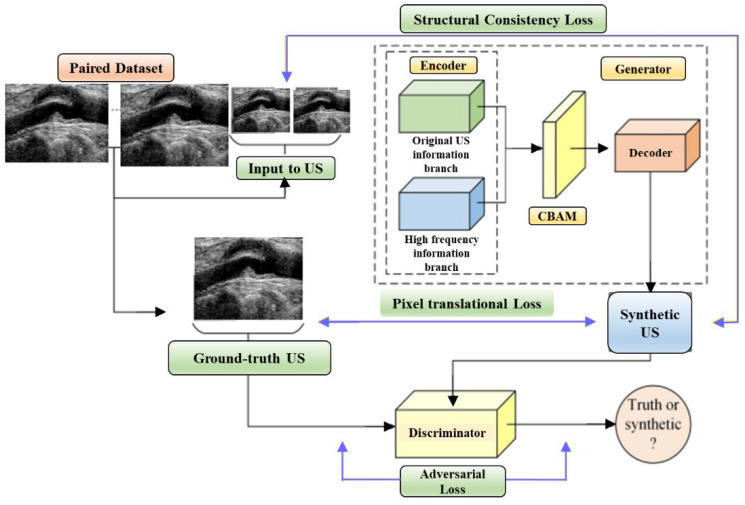
Multilevel Structure-Preserved Generative Adversarial Network (MSP-GAN) architecture.

The limited generalizability of IVUS analysis methods, due to the wide variety of IVUS datasets, is tackled through domain adaptation strategies. Variations in imaging devices, frequencies, and other factors lead to differences in grey distributions, textures, and more among IVUS datasets. Segmentation models designed for the target domain (TD) often fail to generalize to the source domain (SD). Previous approaches required designing a new technique for each SD (re-training DL networks), which is labor-intensive. To address this, domain adaptation is introduced, aiming to translate SD images into realistic TD images (Figure 12). However, current domain adaptation models struggle with preserving intravascular structures due to the complex pathology and low contrast in IVUS images. The challenges of structural preservation in IVUS domain adaptation include (i) the diverse morphologies of vessels, tissues, and artifacts in IVUS image datasets, which can cause the translation process to lose its original status or generate unknown elements.

Therefore, preserving global pathological information is challenging. (ii) Because intravascular structures have limited differentiability and hazy boundaries, it is challenging to guarantee local anatomical consistency between the translated and original pictures. (iii) The absence of corresponding real TD images as reference labels means there is no pixel-wise constraint to prevent distortions of fine structures. Xia et al. [52] introduced MSP-GAN for transferring IVUS domains while preserving intravascular structures. Building upon the generator–discriminator baseline, MSP-GAN incorporates the transformer. Domain adaptation models primarily use generative adversarial networks (GANs), which transfer styles through a loss of adversarial type and preserve content through a content constraint. MSP-GAN enables data-specific IVUS analysis methods to be generalizable across different IVUS datasets. Images adapted using MSP-GAN maintain high anatomical consistency with source images. MSP-GAN employs three innovative techniques for multilevel structural preservation. Table 1 provides a comparative analysis of various IVUS architectures, detailing the advantages, disadvantages, performance metrics, and specific applications of each technique.

## 4. Critical Discussion

### 4.1. Principal Findings

IVUS imaging provides essential cross-sectional views of coronary artery walls for an accurate CVD diagnosis. Manual segmentation is time consuming, labor intensive, and prone to errors. This has spurred significant research into automated segmentation methods, categorized into four main approaches. (i) Traditional methods like thresholding, active contours, and graph-based techniques rely on mathematical models to identify tissue boundaries but often struggle with IVUS image complexity and variability [36]. For example, the Kermani et al. [60] approach effectively detects borders accurately, while the Giannoglou et al. [56] model offers automated segmentation. (ii) ML advancements provide robust and adaptable solutions. Methods like MRF incorporate spatial context, while RF and SVMs use statistical models and ensemble learning to improve accuracy and handle variability in IVUS images. (iii) DL, particularly CNNs, has revolutionized IVUS segmentation. CNNs, such as UNet and its variants, enhance performance by learning hierarchical features from raw data. Advanced architectures like MFA-UNet and IVUS-UNet++ improve accuracy and efficiency by incorporating multi-scale feature aggregation. (iv) Borrowed from natural language processing, attention mechanisms and transformers capture long-term dependencies and contextual information, crucial for IVUS segmentation. Models like the POST [41] and MSP-GAN [52] demonstrate significant improvements in segmentation accuracy by incorporating temporal context, generative adversarial learning, and domain adaptation strategies.

### 4.2. Benchmarking

In the realm of IVUS imaging, various segmentation methods have been developed to enhance the accuracy and efficiency of diagnosing coronary artery disease. Traditional methods, such as conventional techniques proposed by Hammouche et al. [58], utilize a 3D segmentation algorithm with a helical active contour to reduce the effect of ring-down artifacts. Their approach demonstrated high precision with a lumen detection accuracy of 99.42% and a minimal mean absolute error of 0.272 mm as shown in Table 2. Similarly, Giannoglou et al. [56] introduced an active contour model initialized through morphological characteristic analysis. This approach primarily focused on segmenting the lumen-wall and media-adventitia borders, demonstrating a 96% reduction in analysis time compared to manual segmentation. While computationally efficient, this method still depends on well-defined edges, which can be challenging in cases with plaque deposits or noisy IVUS images.

Advancing beyond traditional methods, ML-based techniques have incorporated data-driven strategies for improved segmentation performance. Wang et al. [96] refined the traditional snake algorithm by integrating an extended structure tensor for automatic contour extraction. Their method enhanced edge detection accuracy by employing Otsu thresholding, morphological operations, and connected component labeling. Despite these improvements, the method remains dependent on feature engineering, limiting its adaptability to highly complex IVUS datasets. Another significant contribution in ML-based segmentation came from Vercio et al. [61], who combined support vector machines (SVM) with random forests (RF) to detect morphological structures, followed by a deformable contour model to refine the segmentation of the lumen-intima (LI) and media-adventitia (MA) interfaces. This approach achieved a high Dice similarity coefficient (DSC) of 0.91 for LI and 0.94 for MA, showcasing its effectiveness in distinguishing arterial layers. However, its reliance on feature selection can lead to suboptimal generalization across diverse IVUS datasets.

With the advent of DL, particularly convolutional neural networks CNNs and transformer-based architectures, segmentation techniques have achieved higher accuracy and robustness. Liu et al. [69] introduced a scale-aware multi-path processing (SMP) method, which effectively preserves complementary information across adjacent scales. Their model achieved a DSC of 0.96 for lumen and 0.97 for MA, significantly improving segmentation consistency. The main strength of this approach lies in its ability to address scale-dependent interference, but it requires extensive computational resources, limiting its feasibility in real-time clinical applications. Similarly, Bargsten et al. [78] employed capsule networks, which leverage spatial relationships between image features to enhance performance on small datasets. This method achieved an accuracy of 94.59% in lumen segmentation, outperforming conventional CNNs in terms of spatial awareness. However, capsule networks are computationally intensive, making them challenging to deploy in resource-constrained settings.

The proposed study extends the frontier of IVUS segmentation by employing a transformer- and attention-based architecture. This method capitalizes on the self-attention mechanism, allowing it to capture global contextual dependencies in IVUS images. Unlike CNN-based models, which primarily focus on local features, transformers can effectively model long-range spatial relationships, leading to enhanced segmentation accuracy and robustness. The study was conducted on 39 patients with 500 IVUS scans, demonstrating a promising balance between computational efficiency and segmentation accuracy. However, despite its advantages, transformer models typically require large amounts of training data, and fine-tuning remains essential for domain-specific applications.

Overall, the progression from conventional segmentation techniques to ML- and DL-based approaches reflects a significant leap towards more accurate, automated, and scalable IVUS analysis methods. Each method comes with distinct strengths and limitations, highlighting the continuous need for innovation in medical image segmentation to enhance diagnostic accuracy and clinical applicability in cardiovascular imaging.

### 4.3. A Special Note on Transformers in Coronary Artery Segmentation

Transformers have emerged as a powerful tool in coronary artery segmentation, particularly in the analysis of IVUS imaging [133]. Their key innovation, the self-attention mechanism, allows transformers to capture long-range dependencies and relationships between different parts of an image, making them highly effective in handling complex medical imaging tasks. In the context of coronary artery segmentation, this capability is essential for accurately identifying and segmenting intricate structures such as arterial walls, plaques, and other cardiovascular features that are critical for diagnosing and assessing cardiovascular diseases (CVD) [134,135]. One of the standout advantages of transformers is their ability to model both local and global image features simultaneously [109]. This is particularly important in IVUS imaging, where arterial structures can be difficult to distinguish due to noise, variability in image quality, and overlapping features. By utilizing self-attention mechanisms, transformers can focus on the most relevant regions of an image, while still capturing the broader context, which is vital for distinguishing between structures that may appear similar at the local level but have different overall characteristics. This capability enhances the precision of segmentation, particularly in detecting subtle anatomical details critical for treatment planning. Transformers also integrate well with CNNs in hybrid models, combining the strengths of both architectures [136,137,138]. CNNs are highly effective at extracting detailed local features, while transformers provide a more comprehensive view of the image, handling complex spatial relationships and dependencies. This hybrid approach has led to significant improvements in segmentation accuracy, particularly for challenging cases such as stenosis or calcified regions within the arteries, where local and global information must be processed together to achieve reliable results [139,140,141]. Another important application of transformers in IVUS segmentation is their role in domain adaptation, where they help address the variability in datasets from different imaging sources or devices. Transformers, when combined with generative adversarial networks (GANs) [142,143,144,145,146] or other domain adaptation techniques, help preserve the critical structural information of the coronary arteries, ensuring that segmentation models perform well across different data sources. This ability to generalize across datasets is crucial for real-world clinical applications, where variability in imaging quality and protocols is common [141].

In summary, transformers have made a significant impact on the field of coronary artery segmentation, particularly through their ability to model complex, multi-scale structures, and their adaptability in hybrid architectures. Their contributions to improving segmentation accuracy, robustness, and generalizability position them as a key technology in the future of IVUS imaging and cardiovascular disease diagnosis. While it is technically sound, it is however important to see how the economics of AI using attention and transformers will help in the diagnosis and treatment for CAD [147].

### 4.4. A Special Note on Clinical Relevance and Integration of Transformer-Based IVUS Segmentation into Clinical Practice

The integration of transformer-based segmentation models into IVUS analysis has the potential to revolutionize cardiovascular diagnostics by enhancing precision, reproducibility, and automation in CAD assessments [148]. Unlike traditional segmentation methods, which are often dependent on manual annotation and prone to inter-observer variability, transformer models consistently delineate LI and MA boundaries with higher accuracy, leading to improved measurements of arterial wall thickness and plaque burden. This level of precision is crucial for early detection of atherosclerosis, facilitating timely intervention before significant stenosis develops. Additionally, by minimizing segmentation errors and refining plaque quantification, transformer-based models contribute to more reliable risk stratification in CAD patients, aiding clinicians in developing personalized treatment strategies. The ability of these models to standardize IVUS interpretation across different clinical settings ensures a more objective and data-driven approach to disease assessment, reducing diagnostic variability and improving patient outcomes.

From a clinical workflow perspective, the adoption of transformer-based IVUS segmentation can streamline decision-making in interventional cardiology, particularly in guiding procedures such as angioplasty and stent placement [118]. By providing automated and real-time segmentation outputs, these models enable faster and more informed procedural planning, reducing dependency on manual delineation and enhancing overall efficiency in catheterization labs. Furthermore, their integration with multi-modal imaging (e.g., optical coherence tomography and coronary computed tomography angiography) can offer a more comprehensive visualization of arterial pathology, improving diagnostic confidence. Additionally, incorporating explainability mechanisms [109], can enhance clinical trust and transparency, ensuring that AI-driven segmentation aligns with expert interpretations. However, despite their potential, challenges remain for real-world deployment, particularly in resource-limited settings where computational infrastructure and access to large, annotated datasets for model training may be constrained. Addressing these barriers through model optimization, cloud-based implementations, and domain adaptation techniques will be critical in ensuring equitable access to AI-driven IVUS segmentation, ultimately advancing precision medicine in cardiovascular care.

### 4.5. Strengths: Technological Advancements and Integration

The presented manuscript highlights the importance of accurate coronary artery wall segmentation in IVUS imaging for diagnosing CVD. It points out the challenges of manual segmentation, including its time-consuming nature and potential for errors. The overview of automated methods, from traditional image processing to advanced ML and DL techniques, offers a comprehensive look at the current state of IVUS segmentation research. Categorizing methods into conventional, ML, DL, and attention/transformer methods provides a clear structure [12]. The detailed explanations of techniques like thresholding, active contours, MRF, and RF are insightful. Recent advancements in DL and transformer-based methods showcase improvements in accuracy and efficiency [47,109,149]. The dense technical language and numerous citations might be challenging for non-experts. The section could benefit from a more focused discussion on the clinical implications and real-world impact of these techniques. A deeper exploration of the limitations and trade-offs of conventional and deep learning methods would provide a more balanced view.

The incorporation of real-world case studies in clinical settings highlights the practical benefits of automated IVUS segmentation techniques, showcasing their potential to improve diagnostic accuracy and patient outcomes. Integrating multi-modal imaging data [150,151,152,153], such as combining IVUS with modalities like optical coherence tomography (OCT) or magnetic resonance imaging (MRI), can enhance segmentation accuracy by leveraging complementary information from different sources. Such approaches demonstrate how diverse data streams can work together to overcome limitations inherent to single-modal imaging.

Hybrid methods that combine traditional image processing, machine learning, and deep learning offer a holistic approach to advancing IVUS segmentation. These methods enable precise delineation of complex anatomical structures, such as vessel walls, plaques, and lumen boundaries, by harnessing the strengths of each computational paradigm. By integrating domain knowledge and algorithmic sophistication, these approaches pave the way for robust and interpretable segmentation systems.

Transformer-based architectures, such as Swin UNet and TransUNet, represent a promising avenue for the future of IVUS segmentation. These models integrate global self-attention mechanisms with hierarchical and local feature extraction capabilities, providing the potential to effectively process high-resolution IVUS images. Swin UNet’s shifted window-based self-attention mechanism is well-suited for capturing multi-scale contextual information, while TransUNet combines CNNs for low-level feature extraction with transformers for global dependency modeling. This hybrid architecture may address challenges like artifact interference and enable precise boundary delineation. When used with complementary imaging modalities like OCT or MRI, these models could significantly enhance the robustness and accuracy of IVUS segmentation.

### 4.6. Weaknesses: Limitations in Data, Generalization, and Practicality

While deep learning and transformer-based architectures have demonstrated substantial improvements in IVUS segmentation [154,155], several challenges remain that hinder their widespread clinical adoption. One of the most significant limitations is the dependency on large, high-quality, annotated datasets, which are scarce in IVUS imaging due to the time-intensive nature of expert annotation. Transformer-based models, in particular, require extensive data to capture spatial and contextual relationships effectively, yet the availability of publicly accessible IVUS datasets remains limited [156]. This data scarcity contributes to issues such as overfitting and poor generalization, particularly when models are trained on small and homogeneous datasets. Moreover, the lack of standardized benchmark datasets restricts the ability to fairly compare different segmentation approaches, making it difficult to establish state-of-the-art performance in this domain.

Another critical challenge is the variability across different IVUS scanners and imaging protocols, which leads to inconsistencies in image contrast, resolution, and noise characteristics [157]. Since IVUS devices from different manufacturers employ distinct acquisition settings, deep learning models trained on one dataset often fail to generalize effectively to data from other scanners. Transformer-based architectures, while powerful in capturing long-range dependencies, are highly sensitive to shifts in image distribution, making domain adaptation a crucial but unresolved issue. Although methods such as self-supervised learning and transfer learning offer potential solutions, existing approaches do not fully mitigate the impact of scanner-dependent variations, limiting model applicability in diverse clinical settings.

Furthermore, computational complexity and model interpretability remain major obstacles in deploying transformer-based models for IVUS segmentation in real-time clinical workflows. Compared to CNNs, transformers require significantly higher computational resources, making them impractical for use in resource-constrained environments such as point-of-care applications. Additionally, the lack of transparency in how transformer models process and segment IVUS images poses concerns regarding their reliability in clinical decision-making. Without explainable AI (XAI) mechanisms, it is challenging for clinicians to validate and trust the segmentation outputs, particularly in high-stakes applications like coronary artery disease diagnosis and treatment planning.

### 4.7. Extensions: Future Research and Clinical Implementation

Future research should focus on addressing these limitations to develop clinically viable, efficient, and interpretable transformer-based IVUS segmentation models. A key priority is clinical validation through multi-center studies to evaluate model robustness across different patient populations and imaging conditions. Ensuring that AI-driven segmentation methods perform consistently across datasets acquired from various scanners will be essential for regulatory approval and real-world deployment. Additionally, self-supervised learning, domain adaptation, and federated learning should be explored to improve model generalization while minimizing biases introduced by small or imbalanced datasets. These approaches can enable models to adapt dynamically to new imaging environments, reducing performance discrepancies between datasets.

To enhance computational efficiency, lightweight transformer architectures tailored for IVUS segmentation should be developed. Methods such as MobileViT, Swin UNet, and hybrid CNN–transformer models could help reduce computational overhead while maintaining high segmentation accuracy. Furthermore, integrating multi-modal imaging data, including IVUS, OCT, and MRI, can provide complementary anatomical and functional insights, further improving segmentation precision. Another crucial avenue for future research is the incorporation of XAI techniques, such as attention heatmaps, saliency maps, and uncertainty quantification, to enhance the interpretability of transformer-based models. By making segmentation decisions more transparent and explainable, these techniques can help bridge the gap between deep learning advancements and clinical adoption.

Finally, the development of public benchmark datasets and standardized evaluation protocols is imperative to facilitate the reproducibility and fair comparison of segmentation models. Establishing comprehensive datasets with diverse patient populations and scanner types will drive progress in the field by enabling robust performance evaluations. Future work should also focus on defining clinically relevant metrics that go beyond traditional performance measures like Dice similarity and the Jaccard index, incorporating real-world usability factors such as segmentation speed, model confidence, and clinical utility. By addressing these research directions, transformer-based architectures can be refined into reliable, efficient, and interpretable solutions, ultimately advancing the role of AI in coronary artery disease diagnosis and treatment planning.

## 5. Conclusions

In conclusion, IVUS imaging is crucial for accurate CVD diagnosis, but manual segmentation is time consuming and error prone. Automated segmentation methods have been developed to improve efficiency and accuracy, with traditional techniques providing the foundation. ML advancements, such as MRF, RF, and SVMs, offer robust solutions by incorporating spatial context and ensemble learning. Deep learning, particularly CNNs like UNet and its variants, has revolutionized IVUS segmentation by learning hierarchical features from raw data.

Advanced architectures like MFAUNet and IVUS-UNet++ further enhance accuracy and efficiency. Attention mechanisms and transformers have shown promise in capturing long-term dependencies and contextual information. Models like POST and MSP-GAN demonstrate significant improvements by incorporating temporal context and generative adversarial learning. The evolution of automated segmentation methods has significantly improved IVUS imaging in CVD diagnosis, and future research should continue to refine these methods for clinical applicability and effectiveness.

## Figures and Tables

**Table 1:** Comparative analysis of various IVUS architectures.

Technique	Advantages	Disadvantages	Performance	Specific Application
Conventional Techniques				
Thresholding	Effective in removing catheter artifacts, sequential border estimation, ad hoc mechanism for discontinuous borders	May not perform well with complex and variable IVUS images	Jaccard measure: 0.84 ± 0.07 for Lumen, 0.82 ± 0.11 for MA border	Lumen-intima and media-adventitia (MA) borders segmentation
Active Contours	Fully automated, fast, and adaptive to the shape of the object	Sensitive to initialization, may get stuck in local minima	96% reduction in analysis time compared to manual segmentation	Lumen and MA boundary segmentation
Machine Learning Techniques				
Markov Random Field	Incorporates spatial context, robust segmentation	Designing MRF and defining appropriate potential functions can be challenging, high computational cost	Not specified	Calcified plaque detection
Random Forest	Captures non-linear relationships, handles high-dimensional data well, provides feature importance insights	May overfit with noisy data, less interpretable than simpler models	Not specified	Identifying specific morphological structures within vessel walls
Deep Learning Techniques				
Non-UNet—Scale Mutualized Perception	Preserves complementary information from adjacent scales, distinguishes objects with similar local features	Complex architecture, may require large amounts of data for training	Not specified	Vessel boundary segmentation
Non-UNet—CSDN	Efficient segmentation, treats shallow and deep networks separately for high accuracy and efficiency	Complex architecture, may require large amounts of data for training	Not specified	Real-time segmentation
UNet and its variants (MFA-UNet)	Improves feature fusion and information retention, enables context retrieval from spatial-temporal perspectives	Complex architecture, may require large amounts of data for training	Optimized using Focal Tversky loss to address data imbalance	IVUS scan segmentation
UNet and its variants IVUS-UNet++	More effectively captures fine-grained details of the foreground objects, uses feature pyramid network for multi-scale feature utilization	Complex architecture, may require large amounts of data for training	Best JM and HD for both lumen and MA border compared to UNet++ and IVUS-Net	Lumen and MA border segmentation
Attention and Transformer-based Methods				
POST-IVUS	Accurate segmentation of vessel walls in IVUS images, mimics cardiologists’ perceptual organization principle	Complex architecture, may require large amounts of data for training	Integrated into QCU-CMS1 software for automatic IVUS image segmentation	IVUS image segmentation
MSP-GAN	Preserves intravascular structures during domain adaptation, uses transformers for global pathology information preservation	Complex architecture, may require large amounts of data for training	Ensures local structures correspondence between source and translated images	IVUS domain adaptation

**Table 2:** Benchmarking table. ✓ indicates the presence or use of the specified feature, while ✗ denotes its absence.

C0	C1	C2	C3	C4	C5	C6	C7	C8	C9
SN	Authors	NOF	Type of Data	Architecture Used	Attention	Transformer	#Patients/ #Images	CV	Results
R1	Hammouche et al. [58] (2019)	10	Image	Helical active contour	✗	✗	144/510 497/638	K5	Lumen detection accuracy of 99.42% and a minimal mean absolute error of 0.272 mm.
R2	Giannoglou et al. [56] (2006)	14	Image	Active contour model	✗	✗	97/970	K5	The study demonstrated a 96% reduction in analysis time compared to manual segmentation.
R3	Wang et al. [96] (2021)	17	Image	ML	✗	✗	379, 300	K10	Enhanced traditional snake algorithm, Otsu thresholding, morphological operations, and connected component labeling were incorporated
R4	Vercio et al. [61] (2019)	14	Image	SVM with RF	✓	✗	800	-	High Dice similarity coefficient (DSC) of 0.91 for LI and 0.94 for MA.
R5	Liu et al. [69] (2022)	29	Image	SMP	✓	✓	378	-	DSC of 0.96 for the lumen and 0.97 for the MA
R6	Bargsten et al. [78] (2021)	20	Image	Capsule Network	✓	✓	-	-	Accuracy of 94.59% in lumen segmentation.
R7	Proposed Study	39	Point	Transformers and Attention	✓	✓	500	K5	

## Data Availability

Data are not available due to the proprietary nature of this study.

## Table for Acronyms (SN: Serial Number; Abbn: Abbreviations)

**SN**	**Abbn **	**Description**	**SN**	**Abbn **	**Description**
1	ACS	Acute Coronary Syndrome	43	MCC	Matthews Correlation Coefficient
2	CAD	Coronary Artery Disease	44	AUC	Area Under the Curve
3	CVD	Cardiovascular Disease	45	ROC	Receiver Operating Characteristic
4	CT	Computed Tomography	46	PR	Precision-Recall
5	DL	Deep Learning	47	FPS	Frames Per Second
6	IVUS	Intravascular Ultrasound	48	GPU	Graphics Processing Unit
7	LI	Lumen-Intima	49	CPU	Central Processing Unit
8	MA	Media-Adventitia	50	mAP	Mean Average Precision
9	MRI	Magnetic Resonance Imaging	51	PA	Pixel Accuracy
10	OCT	Optical Coherence Tomography	52	mIoU	Mean Intersection over Union
11	PRISMA	Preferred Reporting Items for Systematic Reviews and Meta-Analyses	53	FWIoU	Frequency Weighted Intersection over Union
12	SDL	Solo Deep Learning	54	TP	True Positive
13	HDL	Hybrid Deep Learning	55	TN	True Negative
14	CNN	Convolutional Neural Network	56	FP	False Positive
15	CRF	Conditional Random Field	57	FN	False Negative
16	FAM	Feature Aggregation Module	58	PPV	Positive Predictive Value
17	BConvLSTM	Bi-directional Convolutional Long Short-Term Memory	59	NPV	Negative Predictive Value
18	MFAUNet	Multi-scale Feature Aggregated UNet	60	FDR	False Discovery Rate
19	UNet++	UNet Plus Plus	61	FOR	False Omission Rate
20	IoU	Intersection over Union	62	MSE	Mean Squared Error
21	JM	Jaccard Measure	63	RMSE	Root Mean Squared Error
22	HD	Hausdorff Distance	64	MAE	Mean Absolute Error
23	GAN	Generative Adversarial Network	65	RAE	Relative Absolute Error
24	MSP-GAN	Multilevel Structure-Preserved Generative Adversarial Network	66	RSE	Relative Squared Error
25	SMC	Super pixel-wise Multi-scale Contrastive Constraint	67	R2	Coefficient of Determination
26	TF	Temporal Constraining and Fusion	68	Adj. R2	Adjusted R-squared
27	STR UNet	Selective Transformer Recurrent UNet	69	AIC	Akaike Information Criterion
28	MIC	Maximum Intensity Curve	70	BIC	Bayesian Information Criterion
29	RMM	Rayleigh Mixture Model	71	LOO-CV	Leave-One-Out Cross-Validation
30	MRF	Markov Random Field	72	K-fold CV	K-fold Cross-Validation
31	FCM	Fuzzy C-Means	73	Lasso	Least Absolute Shrinkage and Selection Operator
32	HMRF	Hidden Markov Random Field	74	Ridge	Ridge Regression
33	SVM	Support Vector Machine	75	Elastic Net	Elastic Net Regression
34	RF	Random Forest	76	PCA	Principal Component Analysis
35	FODPSO	Fractional-order Darwinian Particle Swarm Optimization	77	t-SNE	t-Distributed Stochastic Neighbor Embedding
36	PAD	Percentage of Area Difference	78	UMAP	Uniform Manifold Approximation and Projection
37	HSD	Hausdorff Surface Distance	79	NMF	Non-negative Matrix Factorization
38	Dice	Dice Coefficient	80	ICA	Independent Component Analysis
39	Precision	Precision Score	81	SVD	Singular Value Decomposition
40	Recall	Recall Score	82	LLE	Locally Linear Embedding
41	F1	F1 Score	83	ISOMAP	Isometric Mapping
42	Kappa	Cohen’s Kappa Score	84	MDS	Multidimensional Scaling
